# Immunostimulatory Motifs Enhance Antiviral siRNAs Targeting Highly Pathogenic Avian Influenza H5N1

**DOI:** 10.1371/journal.pone.0021552

**Published:** 2011-07-01

**Authors:** Cameron R. Stewart, Adam J. Karpala, Sue Lowther, John W. Lowenthal, Andrew G. Bean

**Affiliations:** Infection and Immunity, The Commonwealth Scientific and Industrial Research Organisation Australian Animal Health Laboratory, Geelong, Victoria, Australia; University of Georgia, United States of America

## Abstract

Highly pathogenic avian influenza (HPAI) H5N1 virus is endemic in many regions around the world and remains a significant pandemic threat. To date H5N1 has claimed almost 300 human lives worldwide, with a mortality rate of 60% and has caused the death or culling of hundreds of millions of poultry since its initial outbreak in 1997. We have designed multi-functional RNA interference (RNAi)-based therapeutics targeting H5N1 that degrade viral mRNA via the RNAi pathway while at the same time augmenting the host antiviral response by inducing host type I interferon (IFN) production. Moreover, we have identified two factors critical for maximising the immunostimulatory properties of short interfering (si)RNAs in chicken cells (i) mode of synthesis and (ii) nucleoside sequence to augment the response to virus. The 5-bp nucleoside sequence 5′-*UGUGU*-3′ is a key determinant in inducing high levels of expression of IFN -α, -β, -λ and interleukin 1- β in chicken cells. Positioning of this 5′-*UGUGU*-3′ motif at the 5′- end of the sense strand of siRNAs, but not the 3′- end, resulted in a rapid and enhanced induction of type I IFN. An anti-H5N1 avian influenza siRNA directed against the PB1 gene (PB1-2257) tagged with 5′-*UGUGU*-3′ induced type I IFN earlier and to a greater extent compared to a non-tagged PB1-2257. Tested against H5N1 *in vitro*, the tagged PB1-2257 was more effective than non-tagged PB1-2257. These data demonstrate the ability of an immunostimulatory motif to improve the performance of an RNAi-based antiviral, a finding that may influence the design of future RNAi-based anti-influenza therapeutics.

## Introduction

Since the first reports of virus transmission from poultry to humans in 1997, highly pathogenic avian influenza (HPAI) H5N1 virus has spread throughout much of Asia, Europe, the Middle East and Africa [1]. Cases of H5N1 in domestic poultry and water birds have been reported in over 40 countries since 2003, with the death or preventative culling of more than 200 million chickens causing devastation amongst poultry industries [2], particularly in South-East Asia. During 2004, widespread outbreaks of H5N1 occurred throughout Asia, where the virus crossed the species barrier to infect humans [3]. Since then there have been almost 500 confirmed human cases of H5N1 infection reported in 12 countries [4]. Furthermore, the 60% human mortality rate of H5N1 is alarming when compared to the approximate 2% mortality rate of seasonal influenza A [5]. H5N1 remains a significant threat of becoming a pandemic human disease, a scenario tied closely to global outbreaks in poultry. Therefore, novel therapies to combat zoonotic viruses like H5N1 are urgently needed. While vaccines against H5N1 are available, the lack of strain-specific vaccines during early-stage outbreaks creates a demand for alternative therapies. Compounding this is the shortcomings of existing antivirals and their loss of utility due to the emerging viral resistance to classes of neuraminidase (NA) and M2 ion channel inhibitors [6].

RNA interference (RNAi), the natural cellular pathway in which dsRNA input sequence is used to degrade target mRNA, is the basis for many therapeutics currently being developed against major human diseases (reviewed in [7], [8]). The exquisite specificity of RNAi-mediated gene silencing, coupled with a potential to silence virtually any gene, makes RNAi an attractive basis for therapeutic design. Current clinical trials of RNAi-based therapeutics include treatments against several viral diseases, such as respiratory syncytial virus and hepatitis B [9]. However it is well-established that select RNAi molecules can trigger off-target pro-inflammatory and antiviral cytokines which in many cases cause unwanted side effects [10]. This phenomenon, known as immunostimulatory RNAi (isRNAi), may derive from the evolution of RNAi as an antiviral defence mechanism, as observed in plants [11] and Drosophila [12]. Double-stranded RNA may activate the type I interferon (IFN) pathway as an immune mechanism directed to impact on viral replication. Downstream outcomes of type I IFN production and binding to its receptor include the shutdown of RNA and protein synthesis in virally-infected cells, apoptosis of virally-infected cells and the induction of IFN-stimulated genes (ISGs) that coordinate downstream antiviral measures [13]. Stimulation of innate immune responses by RNAi molecules would therefore appear to not be a random process, and several studies have identified factors that confer immunostimulatory properties to RNA, including nucleoside sequence [14], [15], [16], short hairpin RNA (shRNA) promoters [17], [18] and polymerases used to synthesise short interfering RNAs (siRNAs) [16], [19], [20]. Whilst so called off-target effects of RNAi are unwanted in many instances, anti-viral therapeutics may profit greatly from multiple response pathways directed towards prevention of viral replication.

In this study we have explored the application of isRNAi in chickens with the aim of developing isRNAi antivirals that combats H5N1 by silencing viral genes whilst simultaneously triggering antiviral host immune responses to eradicate the virus. We identify a nucleoside motif that strongly induces type I IFN in chicken cells, and explore the strategy of attaching this motif to siRNAs designed against H5N1. By combining a silencing RNA with a nucleoside immuno-enhancers, we have created a new RNAi molecule that complement and synergise in a double-action antiviral.

## Results

**Figure 1:**
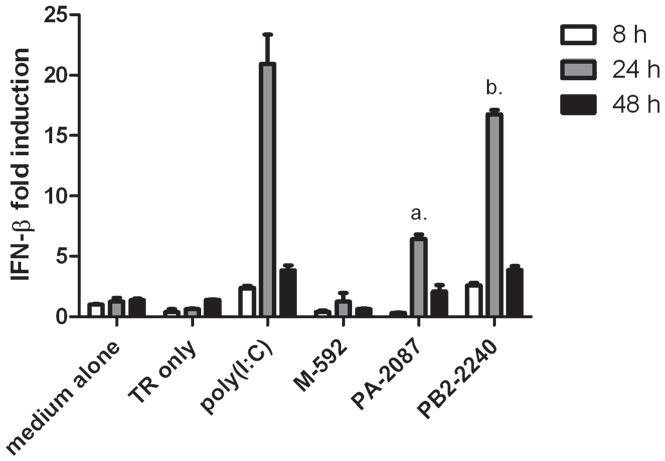
Induction of IFN-β in chicken DF-1 cells by siRNAs. IFN-β mRNA levels induced in DF-1 cells by siRNAs (20 pmol) or polyI:C (0.8 µg). Each value is the mean + standard deviation (3 replicates) and representative of data from 3 experiments. *a* = *p*<0.001 between M-592 and PA-2087 after 8 h. *b* = *p*<0.001 between PA-2087 and PB2-2240 after 8 h.

siRNAs designed against three different conserved regions of the influenza A genome were selected based on their silencing ability (PA-2087 and PB2-2240 [21], M-592 – T. Doran, unpublished data) (Table 1, sequences 1–3). To test the ability of these siRNAs to induce cytokine production in chicken cells, these three siRNAs were synthesized using T7 RNA polymerase and transfected into the immortalized chicken fibroblast cell line, DF-1. Since DF-1 cells have previously been shown to produce high levels of IFN- β in response to the dsRNA mimetic polyI:C [22], they provide a model cell line for assessing the type I IFN response to siRNA. M-592, PA-2087 and PB2-2240 induced IFN-β to differing levels, effectively giving low, moderate and high induction of IFN-β, respectively, relative to induction by polyI:C, with maximum levels observed after 24 h (Figure 1).

**Table 1:** siRNAs used in this study

Sequence #	siRNA	Sequence
1	M-592	5′-CUACAGCUAAGGCUAUGGA-3′ 3′-GAUGUCGAUUCCGAUACCU-5′
2	PA-2087	5′-GCAAUUGAGGAGUGCCUGA-3′ 3′-CGUUAACUCCUCACGGACU-5′
3	PB2-2240	5′-CGGGACUCUAGCAUACUUA-3′ 3′-GCCCUGAGAUCGUAUGAAU-5′
4	PB1-2257	5′-GAUCUGUUCCACCAUUGAA-3′ 3′-CUAGACAAGGUGGUAACUU-5′
5	isPB1-2257	5′-GA**UGUGU**UCCACCAUUGAA-3′ 3′-CUACACAAGGUGGUAACUU-5′
6	Scramble	5′-AAGAGAGCGAGAUCUACAC-3′ 3′-UUCUCUCGCUCUAGAUGUG-5′
7	2Me-isPB1-2257	5′-GA**U[mG]UGU**UCCACCAUUGAA-3′ 3′-CUACACAAGGUGGUAACUU-5′
8	uPB1-2257	5′-**UGUGU**GAUCUGUUCCACCAUUGAA-3′ 3′-ACACACUAGACAAGGUGGUAACUU-5′
9	PB1-2257u	5′-GAUCUGUUCCACCAUUGAA**UGUGU**-3′ 3′-CUAGACAAGGUGGUAACUUACACA-5′

**Figure 2:**
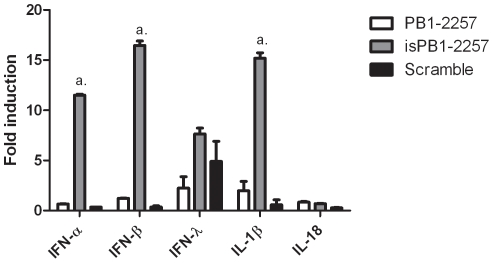
Increased immunostimulatory properties of siRNAs containing *5′-UGUGU-3′* in chicken cells. mRNA levels of cytokines in DF-1 cells induced by PB1-2257, isPB1-2257 and Scramble (20 pmol) after 8 h. Each value is the mean + standard deviation (3 replicates) and representative of data from 3 experiments *a* = *p*<0.001 between isPB1-2257 and both PB1-2257 and Scramble after 8 h.

The ability of distinct sequences to modulate the immunostimulatory properties of siRNAs in mammalian cells was illustrated recently [15] where the induction of pro-inflammatory cytokines was directly related the presence of a 5 bp motif, *5′-UGUGU-3′* . We conducted a bioinformatics search of RNAi sequences targeting influenza A and found that PB1-2257, a 19 bp siRNA targeting the PB1 segment of influenza A contains a motif from base pairs 3–7, *5′-UCUGU-3′* , remarkably similar to *5′-UGUGU-3′* (Table 1, sequence 4). Previous studies have shown PB1-2257 to be an effective silencer of influenza A subtype H1N1 replication [21]. We created a modified version of PB1-2257 (immunostimulatory (is)PB1-2257) where a single substitution at position 4 (C to G) was made, hence creating a *5′-UGUGU-3′* motif (Table 1, sequence 5). An irrelevant siRNA (Scramble) was also synthesized as a negative control by random shuffling of the PB1-2257 sequence (Table 1, sequence 6). isPB1-2257 induced IFN-α, IFN-β, IFN-λ and IL-1β mRNA to a greater extent than PB1-2257 and Scramble after transfection into DF-1 cells (Figure 2). Interestingly, PB1-2257, isPB1-2257 and Scramble induced IL-1β, but not the related cytokine, IL-18.

**Figure 3:**
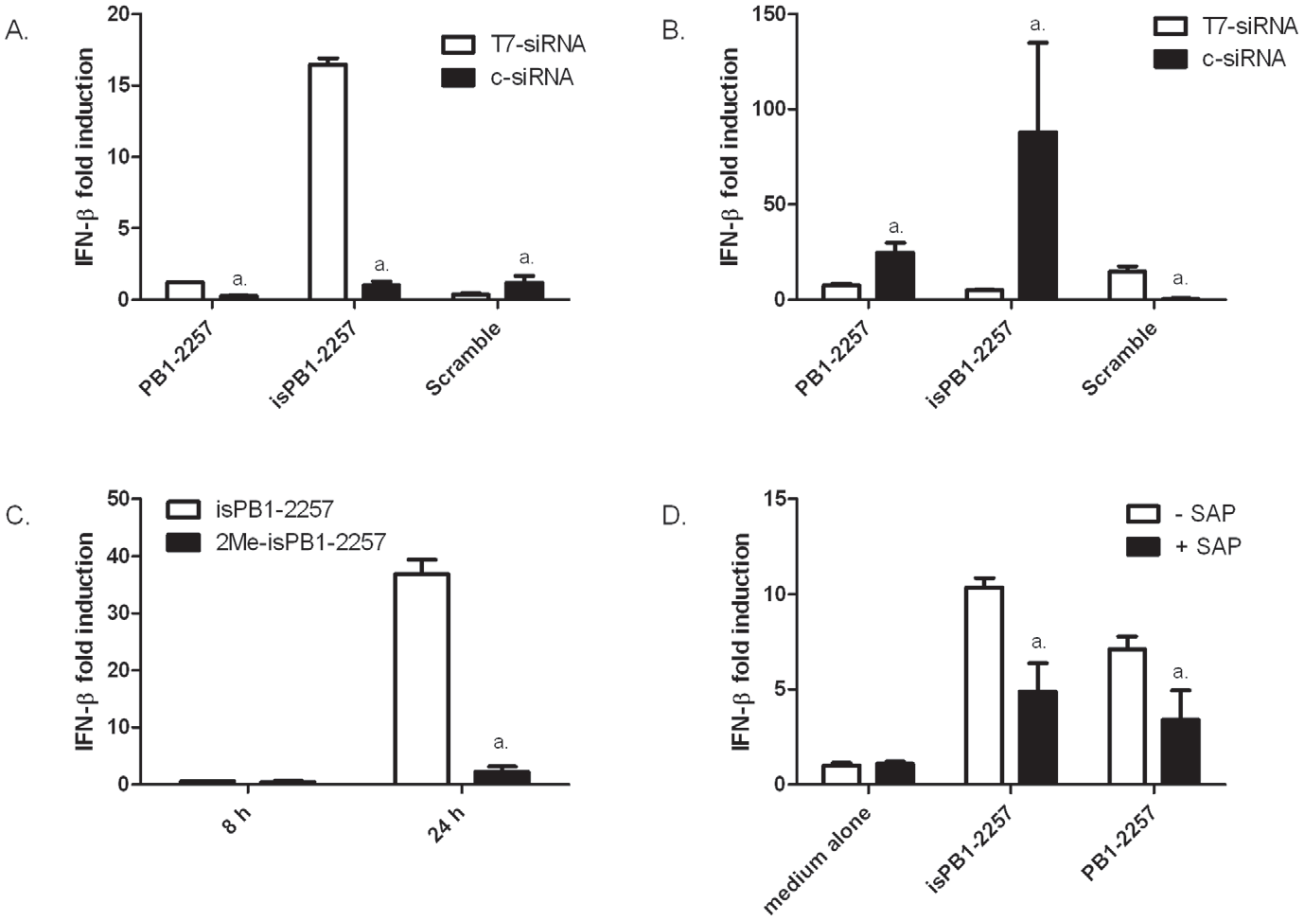
Immunostimulatory properties of T7-siRNAs and c-siRNAs in chicken cells. IFN-β mRNA levels were measured in DF-1 cells transfected with T7-siRNAs (white bars, 20 pmol) or c-siRNAs (black bars, 20 pmol) at (A) 8 h or (B) 24 h. *a* = *p*<0.05 between paired white bars (T7-siRNA) and black bars (c-siRNA). (C) IFN-β mRNA levels in DF-1 cells induced by isPB1-2557 (chemically synthesized, 20 pmol) or 2Me-isPB1-2257 (chemically-synthesized, 20 pmol). *a*. = *p*<0.001 between isPB1-2257 and 2Me-isPB1-2257 at 24 h. (D) IFN-β mRNA levels in DF-1 cells transfected with T7-siRNAs (20 pmol) untreated (white bars) or treated with shrimp alkaline phosphatase (SAP) (black bars). Data shows IFN-β mRNA levels 8 h post-transfection. *a*. = *p*<0.05 between levels of IFN-β in cells transfected with untreated siRNAs or SAP-treated siRNAs. Results are representative of 3 separate experiments.

It has been reported that mode of synthesis can impact the immunostimulatory properties of siRNAs [10]. To test this parameter in our study, chemically-synthesized siRNAs (c-siRNAs) and siRNAs synthesized using T7 RNA polymerase (T7-siRNAs) were transfected into DF-1 cells at identical concentrations and the induced changes in IFN-β mRNA levels were measured. By 8 h post-transfection (Figure 3 A), the T7-isPB1-2257 had induced a large increase in IFN- β. However, this increase was not observed for the c-siRNA variant, nor either form of PB1-2257 or Scramble. At 24 h (Figure 3 B), induction of IFN- β was 10-fold higher for c-isPB1-2257 compared to T7-isPB1-2257. Scramble induced moderate IFN-β expression at 24 h as a T7-siRNA, but not as a c-siRNA. These results demonstrate that for both T7-siRNAs and c-siRNAs, there is a sequence-dependent induction of IFN-β. This induction is more rapid with the additional input of T7 synthesis.

To further probe the role of *5′-UGUGU-3′* in IFN stimulation, a chemically-synthesized derivative of isPB1-2257 was synthesized with a methyl group masking the *5′-UGUGU-3′* motif (Table 1, sequence 7). Masking of the *5′-UGUGU-3′* motif caused an almost total abolition of IFN-β induction by isPB1-2257 at 24 h (Figure 3C). To test the role of 5′ phosphate groups present on siRNAs synthesised by T7 RNA polymerase on immunostimulation, T7-isPB1-2257 and T7-PB1-2257 were treated with shrimp alkaline phosphatase (SAP) to remove 5′ phosphate groups and transfected into DF-1 cells. Compared to untreated controls, levels of IFN-β induced by isPB1-2257 and PB1-2257 were reduced by approximately 40% by phosphatase treatment (Figure 3D). These results confirm the importance of both the *5′-UGUGU-3′* motif and mode of siRNA synthesis on immunostimulation.

**Figure 4:**
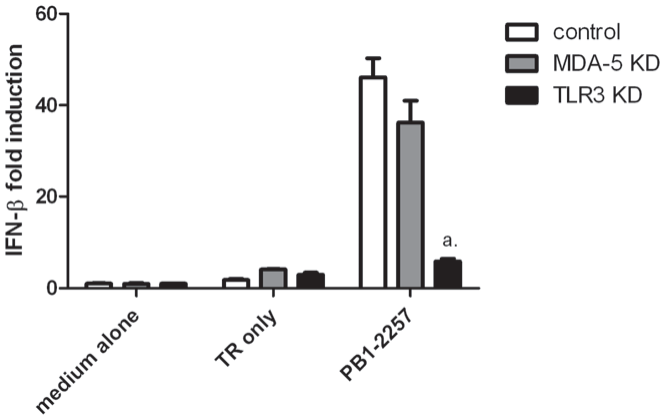
T7-PB1-2257 requires TLR3 but not Mda5 for type I IFN induction in DF-1 cells. Control (white bars), Mda5-knockdown (striped bars) and TLR3-knockdown (black bars) DF-1 cells were transfected with PB1-2257 (T7-siRNA, 20 pmol). mRNA levels of IFN-β were measured by QRT-PCR 24 h after PB1-2257 stimulation. *a* = *p*<0.001 between IFN-β levels induced by PB1-2257 in control cells (white bars) and TLR3 KD cells (black bars). Results are representative of 3 separate experiments.

We next investigated the receptor responsible for inducing type I IFN in response to immunostimulatory siRNAs in chicken cells. Immunostimulatory siRNAs act via Toll-like receptor 7 (TLR7) in mammals [14]. However, QRT-PCR demonstrated that DF-1 cells express almost non-detectable levels of TLR7 (not shown), suggesting that other dsRNA-sensing molecules, such as other TLRs or RIG-like helicases, may recognise PB1-2257. siRNAs were transfected into DF-1 cells to create cell populations knocked down for melanoma-differentiation-associated gene 5 (Mda5) and TLR3. siRNAs to silence chicken TLR3 and chicken Mda5 have been described previously and reduce gene expression levels by >75% [22], [23]. QRT-PCR results confirmed that siRNAs and did not themselves induce significant increases in type I IFN (data not shown). A strong induction of IFN-β in control DF-1 cells was observed 24 h after stimulation with PB1-2257 (Figure 4). This response was reduced by 90% in TLR3 knock down DF-1 cells (p<0.001). In contrast, cells with reduced levels of Mda5 did not show a significant reduction in IFN-β expression.

**Figure 5:**
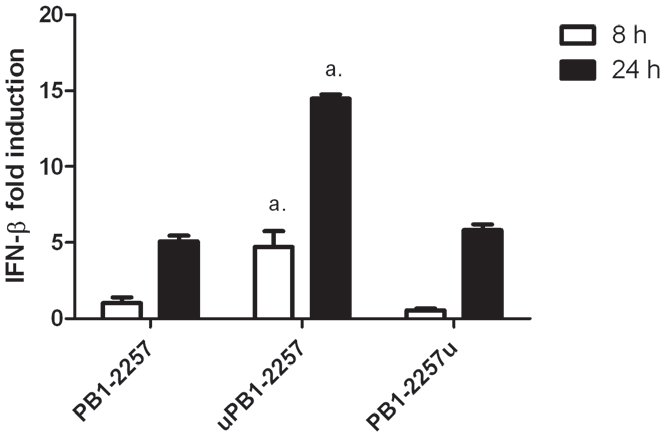
Attaching *5′-UGUGU-3′* to PB1-2257 increases induction of type I IFN. mRNA RNA levels of IFN-β in DF-1 cells transfected with chemically-synthesized PB1-2257, PB1-2257 tagged with *5′-UGUGU-3′* at the 5′ end (uPB1-2257) or at the 3′ end (PB1-2257u) (all 20 pmol) for 8 h or 24 h. *a* = *p*<0.05 between levels of IFN-β induced by uPB1-2257 and PB1-2257. Each value is the mean + standard deviation (3 replicates) and representative of data from 3 experiments.

While results in figure 2 and figure 3 show that isPB1-2257 is more immunostimulatory than PB1-2257, this effect was achieved by altering the PB1-2257 sequence, which is critical for silencing. To create a multi-functional antiviral with both immunostimulatory and silencing properties, we attached *5′-UGUGU-3′* to either the 5′ or the 3′ ends of c-PB1-2257 without disrupting the silencing sequence itself (Table 1, sequence 8 and 9). This modification resulted in a 3-fold enhancement of the immunostimulatory profile for the PB1-2257 variant tagged with the *5′-UGUGU-3′* motif at the 5′ end (uPB1-2257) relative to the un-tagged molecule (Figure 5). In contrast, tagging at the 3′ end (PB1-2257u) appeared to have little enhancing effect.

**Figure 6:**
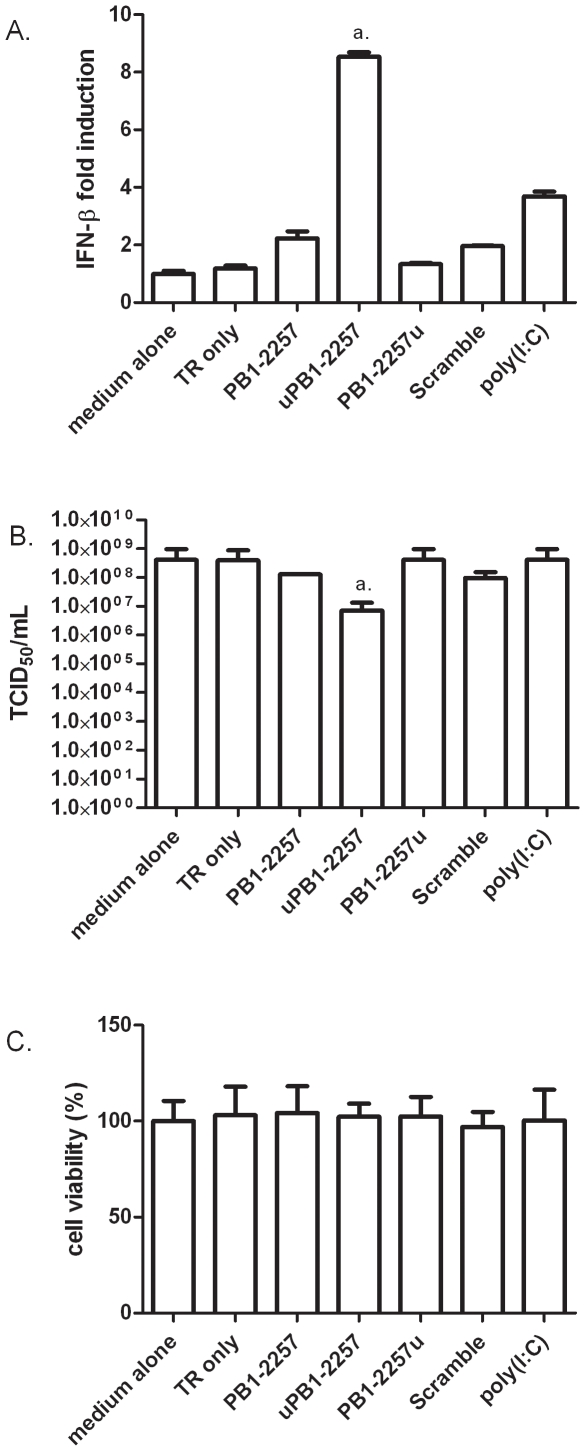
Inhibition of H5N1 influenza virus growth by *5′-UGUGU-3′* -tagged PB1-2257. (A) IFN-β mRNA levels in HD-11 cells transfected with c-siRNAs (all 20 pmol). Data shows IFN-β mRNA levels 8 h post-transfection. *a*. = *p*<0.01 between levels of IFN-β in cells transfected with uPB1-2257 versus PB1-2257. (B) TCID_50_ of H5N1 virus growth in HD-11 cells transfected with c-siRNAs (5 pmol, 96 well plate) or polyI:C (0.1 µg). Cells were transfected with siRNAs and infected with H5N1 virus 24 h later at dilutions of virus of 10^-1^to 10^−7^. Cells were rinsed with fresh medium before the addition of virus. TCID_50_ values were measured 72 h post-infection. Each value is the mean + standard deviation (4 replicates). *a* = *p*<0.05 between virus levels in cell supernatants of cells treated with PB1-2257 or uPB1-2257. (C) Viability of HD-11 cells 24 h post-transfection with c-siRNAs (5 pmol, 96 well plate) as measured by Alamar blue. Results are representative of 3 separate experiments.

We tested siRNA-mediated inhibition of influenza A/Vietnam/1203/2004 (H5N1) *in vitro*. Since DF-1 cells are poor at supporting H5N1 virus replication (data not shown), the immortalised chicken macrophage cell line, HD-11, was used for this experiment. Firstly, we investigated whether *5′-UGUGU-3′* -tagged PB1-2257 showed enhanced immunostimulatory properties in HD-11 cells (Figure 6A). Similar to results observed for DF-1 cells, HD-11 cells transfected with uPB1-2257 showed increased levels of IFN-β at 8 h compared to cells transfected with PB1-2257 or PB1-2257u. When infected with H5N1 virus (Figure 6B), HD-11 cells transfected with uPB1-2257 showed significantly reduced levels of virus compared to cells transfected with PB1-2257 or PB1-2257u. To ensure that siRNA-mediated inhibition of H5N1 virus growth was not due to toxicity, the viability of HD-11 cells transfected with siRNAs was measured using Alamar Blue. At the time of H5N1 infection (24 h post-transfection), cells transfected with siRNAs showed viability levels comparable to untreated cells, (Figure 6C) suggesting that antiviral properties of siRNAs are not related to compromised cell viability.

## Discussion

Highly pathogenic avian influenza (HPAI) H5N1 virus remains a significant pandemic threat. The development of novel antivirals to assist in protection against H5N1 virus will present healthcare industries with a new approach in dealing with an outbreak of viral infection. This study is the first to demonstrate the effective inhibition of highly pathogenic avian influenza by directed immunostimulatory siRNAs. Furthermore, we have identified several factors critical to the immunostimulatory properties of siRNAs in chicken cells. Firstly, the importance of sequence was demonstrated by comparing the immunostimulatory properties of PB1-2257 and isPB1-2257 in DF-1 cells, where a single nucleoside substitution had a large impact on the kinetics and extent of IFN production. In addition, we showed that siRNAs synthesized enzymatically had different immunostimulatory properties to chemically-synthesized sequence-matched variants, demonstrating how the mode of siRNA synthesis influences immunostimulation in the chicken. This difference in immunostimulation between siRNA variants was caused partially by cellular recognition of 5′-triphosphate groups present in T7-siRNAs. Interestingly, a recent report suggested that DF-1 cells do not produce type I IFN in response to 5′-triphosphate siRNAs due to an absence of retinoic acid-inducible gene I protein (RIG-I) in chickens, and that a decreased type I IFN response in chickens in response to viral RNA epitopes may explain the increased susceptibility of chickens to H5N1 virus [24]. However, our findings would suggest that DF-1 cells can produce type I IFN in response to phosphorylated siRNAs and that this process is critically sequence-dependent.

This study provides further evidence that the immunostimulatory properties of siRNAs, for several years considered an unwanted side effect of siRNA research, can be utilised to improve the performance of antiviral siRNAs. This synergistic effect was recently achieved in a mammalian system by introducing a microRNA-like non-pairing uridine-bulge into siRNA passenger strands, resulting in immunostimulatory siRNAs that enhanced protection of human immune cells from Semliki Forest virus [25]. We have harnessed the immunostimulatory properties of the *5′-UGUGU-3′* motif, and illustrated how the differential positioning of the *5′-UGUGU-3′* motif at the 5′ end of PB1-2257 can have a major impact on immunostimulation and subsequent antiviral protection. In chicken cells infected with H5N1, the effect of 5′ tagging lead to a 2-log enhancement of anti-viral activity. Interestingly, a 21 bp siRNA duplex consisting entirely of poly-UG has recently been shown to induce type I IFNs in chicken splenocytes more effectively than siRNAs lacking poly-UG repeats [26]. This result further demonstrates the immunostimulatory properties of siRNAs featuring poly-UG motifs such as *5′-UGUGU-3′* and supports our strategy to enhance PB1-2257 using this motif.

A major finding from this study is that immunostimulation of chicken cells by T7-siRNAs involves TLR3, a result that is perhaps surprising given that immune recognition of short dsRNA has been assigned to TLR7 and TLR8 in other species [27]. However, it is well established that TLR3, while long-associated with the recognition of long (>46 bp) dsRNA, can be activated by RNA duplexes as short as 21 bp [28], [29]. An example of this activation is the design of siRNAs against vascular endothelial growth factor-A as a treatment for age-related macular degeneration [30]. It was subsequently demonstrated that numerous dsRNAs inhibited angiogenesis in a sequence-independent manner by activating TLR3 and the downstream production of IFN-γ and IL-12 [29]. Our results suggest a hitherto unreported role for chicken TLR3 in the recognition of short dsRNA. This is of particular interest given that the apparent lack of RIG-I in the chicken genome would suggest other receptors are responsible for viral dsRNA surveillance. Having already established the role for chicken TLR3 in the recognition of long dsRNA [22], our results suggest that TLR3 plays a dual role in recognition of both long and short dsRNA.

It has recently been shown that RNAi potency and efficacy can be improved by creating siRNAs as longer, 27 b.p. Dicer substrates [31], [32]. One possible explanation for the enhanced anti-viral impact of UGUGU-tagged PB1-2257 is that lengthening the siRNA creates a Dicer substrate with improved potency. However, two factors would suggest that the improved anti-viral properties of UGUGU-tagged PB1-2257 are caused by immunostimulation. Firstly, we created two longer variants of PB1-2257; uPB1-2257 and PB1-2257u. Only for uPB1-2257 did we observe increased IFN production and improved anti-viral properties, suggesting this result was not caused by non-specific lengthening of the siRNA duplex. Secondly, the addition of UGUGU to PB1-2257 created a 24 b.p. duplex – which in theory should be too short to act as a Dicer substrate.

The ability of *5′-UGUGU-3′* to incite antiviral immune responses in different species [15], coupled with the use of siRNAs targeting viral genes rather than host genes, raises the prospect of immunostimulatory siRNAs being developed as therapeutics against a diverse range of viruses across different species. This point is particularly relevant given the ability of several devastating viruses, including swine-origin influenza virus and H5N1, to transmit across species. In addition, the combinational use of siRNAs designed against conserved regions of viral genomes would create therapeutics effective against various virus subtypes. For example, therapeutic siRNAs targeting conserved regions of the influenza A genome would silence H5N1 and H1N1, that have caused recent devastation of poultry and pork industries respectively, and caused human fatalities. As the availability of a strain-specific vaccine is unlikely in the early stages of a virus outbreak, and given the rapid onset of mortality, immunostimulatory siRNAs represent an alternative therapy to combat viral infections.

## Materials and Methods

### siRNAs

siRNAs were synthesized by T7 RNA polymerase using the Ambion Silencer® siRNA Construction Kit according to manufacturer's instructions (Ambion inc, Austin, Texas) with template oligonucleotides purchased from Geneworks (Adelaide, Australia). Chemically-synthesized siRNAs were purchased from Sigma (St Louis, MO). siRNAs used to reduce chicken TLR3 [22] and Mda5 [23]) have been described previously.

### Cell culture

The continuous chicken fibroblast cell line DF-1 (American Type Culture Collection (ATCC), CRL-12203) were maintained in DMEM supplemented with 10% (v/v) heat-inactivated foetal calf serum (FCS), 2 mM L-glutamine, 1.5% (w/v) sodium bicarbonate, 100 U/mL penicillin and 100 µg/mL streptomycin. The continuous chicken macrophage cell line HD-11 was maintained in RPMI medium supplemented with 10% (v/v) FCS, 10 mM HEPES, 2 mM L-glutamine, 100 U/mL penicillin, and 100 µg/mL streptomycin. African green monkey kidney epithelial Vero cells (ATCC CRL-81) were maintained in DMEM supplemented with 10% (v/v) FCS, 100 U/mL penicillin and 100 µg/mL streptomycin. All cells were incubated at 37°C under a 5% CO_2_/95% air atmosphere.

### Transfections

For RNA extraction, DF-1 and HD-11 cells were cultured in 12-well plates (1×10^5^ cells per well) for 24 h before transfection in medium lacking penicillin and streptomycin. Cells were transfected using Lipofectamine 2000 (Invitrogen, Carlsbad, CA) according to manufacturer's guidelines. After 4 h, the transfection medium was changed to growth medium containing penicillin (100 U/mL) and streptomycin (100 µg/mL) and 10% FCS. For viral infections, HD-11 cells were seeded in 96 well plates (6×10^3^ cells per well) for 24 h before transfection in medium lacking penicillin and streptomycin. Cells were transfected using Lipofectamine 2000 according to manufacturer's guidelines. After 4 h, the transfection medium was changed to growth medium containing penicillin (100 U/mL) and streptomycin (100 µg/mL) and 10% FCS.

### RNA isolation, reverse transcription and quantitative real-time PCR

RNA was harvested using Tri-reagent (Sigma) according to manufacturer's instructions. One microgram of extracted RNA was treated with DNase (Sigma) according to manufacturer's instructions and reverse-transcribed to complementary DNA (cDNA) using a Reverse Transcription kit (Promega, Madison WI). Quantitative real-time PCR (QRT-PCR) experiments were conducted to measure cytokine expression levels. All quantification data was normalised against chicken GAPDH. QRT-PCR was performed on an ABI Prism 7700 Sequence Detection System (Applied Biosystems). The comparative threshold cycle (Ct) method was used to derive fold change gene expression. Primers and probes were designed using Primer Express software (Applied Biosystems). Where possible, the probe sets were designed across intron:exon boundaries. PCR cycling was performed as follows: 95°C for 1 min, followed by 40 cycles of 95°C for 15 sec, 61°C for 30 sec and 68°C for 30 sec.

#### Quantitative real-time PCR probes

Probes for the detection of chicken IFN-α, IFN-β, IFN-λ and GAPDH have been described previously [22], [33]. Sequences for detecting chicken IL-1β and chicken IL-18 are shown in Table 2.

**Table 2:** QRT-PCR primers and probes used in this study

Target gene	primer/probe	Sequence (5′ to 3′)	Accession #
chIL1β	F	AGCGGCACCGAGCC	NM204524
	R	GGGTCAGCTCGACGCT	
	Probe	CTTGGCTGGTTTCTCC	
chIL18	F	TCGACATTCACTGTTACAAAACCA	NM204608
	R	ACCTGGACGCTGAATGCAA	
	Probe	CGCGCCTTCAGCAGGGATGC	

### Treatment of siRNAs with shrimp alkaline phosphatase

siRNAs synthesized using T7 RNA polymerase were treated with shrimp alkaline phosphatase (SAP) according to manufacturers guidelines (Promega). Briefly, siRNAs were treated with SAP (1u SAP/µg siRNA) at 37°C for 30 min. Samples were heated to 65°C for 10 min to inactivate SAP.

### Viruses

Influenza A/Vietnam/1203/2004 (H5N1) was passaged in the allantoic fluid of 10-day embryonated specific pathogen-free chicken eggs. H5N1 virus was handled in BSL3 conditions at the CSIRO Australian Animal Health Laboratory (AAHL). Allantoic fluid was harvested, aliquoted and stored at −80°C for inoculations. Virus titer was measured by plaque assays, whereby serial 10-fold dilutions of viruses were incubated on Vero cells for 30 minutes and then cells were overlayed with medium containing 1% agar. Two days after infection, plaques were visualized by staining with crystal violet.

### TCID_50_

10-fold dilutions of H5N1 virus were made in PBS and added to a 96-well tissue culture plate containing Vero cells in growth medium. Plates were incubated for 5 days at 37°C, 5% CO_2_ and scored for cytopathic effect. The infectious titer was calculated by the method of Hawkes [34].

### Cell viability assay

Viability of HD-11 cells was measured using Alamar Blue (Invitrogen) according to manufacturer's guidelines. Briefly, HD-11 cells seeded in 96-well tissue culture plates were incubated with 10 µL Alamar Blue for 4 h at 37°C 5% CO_2_. Sample absorbance was measured at 540 nm and 620 nm using a Biotek EL808 absorbance microplate reader (Biotek, Winooski, VT). Cell viability was calculated by subtracting the 620 nm absorbance values of the cell culture medium alone from 540 nm absorbance values of experimental wells.

### Statistics

The difference between two groups was statistically analysed by Student's *t*-test. A *p*-value of <0.05 was considered significant.
